# Evaluation for radiographic changes in the coxofemoral joint and proximal femur following intramedullary stabilisation of diaphyseal femoral fractures using angle‐stable interlocking nails in skeletally immature patients

**DOI:** 10.1111/jsap.70129

**Published:** 2026-03-29

**Authors:** L. M. Meneghetti, S. R. O'Neill, K. L. Perry

**Affiliations:** ^1^ Michigan State University Veterinary Medical Center East Lansing Michigan USA

## Abstract

**Objectives:**

To evaluate a juvenile dog population that sustained diaphyseal femoral fractures surgically stabilised with an angle‐stable interlocking nail and evaluate for evidence of proximal femoral malformation both periooperatively and at skeletal maturity.

**Materials and Methods:**

Retrospective study of ten skeletally immature dogs sustaining diaphyseal femoral fractures stabilised using an angle‐stable interlocking nail between January 2010 and June 2023. Standardised orthopaedic examination, Canine Brief Pain Inventory and radiographic review were performed at skeletal maturity. Radiographic review included subjective assessment for proximal femoral malformation and objective assessment of anatomic and mechanical angles of alignment in both the frontal and sagittal planes, immediately post‐operatively, perioperatively and at long‐term follow‐up. A Wilcoxon signed rank test was used to assess femoral measurements using the unaffected femur as a control in each dog.

**Results:**

Six of the nine dogs scored 0 on the Canine Brief Pain Inventory, while the remaining three had low scores (maximum of 10/100). No case had evidence of proximal femoral malformation during perioperative follow‐up. Two dogs developed bilateral coxofemoral joint osteoarthritis at skeletal maturity. CORONA1 was the only significantly different measurement when comparing the surgical to the unaffected femur at skeletal maturity.

**Clinical Significance:**

Use of an angle‐stable interlocking nail to stabilise femoral diaphyseal fractures in skeletally immature dogs can be performed without significant risk of developing proximal femoral malformation. Reductions in CORONA1 values may be anticipated due to reducing natural femoral procurvatum. Angle‐stable interlocking nails should not be considered contraindicated for use in canine juvenile diaphyseal femoral fracture repair.

## INTRODUCTION

The literature detailing fractures sustained in companion animals repeatedly cites juveniles as over‐represented (Aithal et al., 1999; Braden et al., 1995; Hunt et al., 1980; Kolata et al., 1974; Kolata & Johnston, 1975; Kumar et al., 2020; Libardoni et al., 2018; Phillips, 1979; Raouf et al., 2019; Talaat et al., 2022). Femoral fractures are common, representing 15% to 38% of all fractures (Kolata & Johnston, 1975; Kumar et al., 2020; Minar et al., 2013; Raouf et al., 2019) and 14.8% to 44% of long‐bone fractures (Aithal et al., 1999; Kolata & Johnston, 1975; Phillips, 1979; Raouf et al., 2019; Talaat et al., 2022). Among femoral fractures, the diaphysis is the most commonly affected site (Aithal et al., 1999; Braden et al., 1995; Hunt et al., 1980; Talaat et al., 2022). Diaphyseal fractures of the femur are often simple; however, in up to 33% of cases severe comminution is reported (Braden et al., 1995; Hunt et al., 1980), likely due to the most frequent aetiology being vehicular trauma (Aithal et al., 1999; Kolata et al., 1974; Kumar et al., 2020; Libardoni et al., 2018; Minar et al., 2013; Phillips, 1979; Raouf et al., 2019).

Accepted practices for diaphyseal femoral fracture stabilisation in skeletally immature patients are limited due to the biomechanical properties of immature bone (Torzilli et al., 1981, 1982). Immature bone is less dense due to its relative porosity (Torzilli et al., 1982), leading to an increased risk of screw pull‐out (Chapman et al., 1996). The use of traditional plate osteosynthesis has been associated with high rates of screw pull‐out in skeletally immature patients due to the construct being overly stiff in comparison with soft juvenile bone (Smith et al., 1996; Vizcaíno Revés et al., 2013). Elastic plate osteosynthesis has been performed successfully in dogs under 5 months of age with excellent results postulated to be due to the inherent elasticity of the construct essentially eliminating the risk of screw pull‐out and favouring robust callus formation (Cabassu, 2001). However, the limitations of this technique are yet to be fully described, and allowing excessive construct elasticity or overloading the implants, such as in comminuted fractures, may result in catastrophic failure (Sarrau et al., 2007).

The use of intramedullary implants may be biomechanically superior to other forms of fixation, as load is placed along the long axis of the implant itself, neutralising bending forces (Beale, 2004). However, the use of intramedullary implants in the femora of skeletally immature patients has essentially been considered contraindicated after one study found that over 87% of cases had at least one area of proximal femoral malformation (PFM) (Black & Withrow, 1979). These included small or malformed femoral heads, short, thin femoral necks, coxa valga and coxofemoral subluxation (Black & Withrow, 1979). Similar effects have also been reported in human children following use of femoral intramedullary nailing (González‐Herranz et al., 1995; Herzog, 1977).

This presents a major obstacle for diaphyseal femoral fracture stabilisation in juvenile patients where substantial implant‐associated loads are anticipated. This may include polytrauma patients, comminuted fractures or cases where soft tissue and vascular compromise may lead to more protracted healing.

The authors have used angle‐stable interlocking nails (AS‐ILN) for select cases of juvenile diaphyseal femoral fracture; specifically, those considered at higher risk of failure should eccentric stabilisation methods be used in isolation. Subjectively, no overt PFM has been observed. Therefore, the aim of this study was to evaluate skeletally immature patients that had an AS‐ILN placed to stabilise a diaphyseal femoral fracture and re‐evaluate them at skeletal maturity to assess clinical function and for any radiographic evidence of PFM. We hypothesised that the affected femur would have no evidence of malformation and that patients would demonstrate no clinical differences when compared to the contralateral unaffected limb at skeletal maturity.

## MATERIALS AND METHODS

### Case selection

The study protocol was approved by the Institutional Animal Care and Use Committee at Michigan State University (approval number: PROTO202200466). Medical records of skeletally immature dogs with diaphyseal femoral fractures stabilised with an AS‐ILN (I‐Loc; BioMedtrix, Whippany, New Jersey) between January 2010 and June 2023 at one institution were compiled. Skeletal immaturity was dictated by the presence of open proximal femoral physes. Skeletal maturity was defined as patients over 1 year of age based on previously reported rates of proximal femoral physeal closure (Sutton et al., 2018). Data including breed, sex, neuter status, age, fracture aetiology, method of nail insertion and bolt configuration were recorded.

The owner of each dog was contacted and informed of study aims and requirements. Inclusion criteria consisted of having open proximal femoral physes at the time of fracture repair, a diaphyseal femoral fracture stabilised using an AS‐ILN and re‐evaluation at skeletal maturity. Patients were included if initial follow‐up was lost prior to clinical union. Patients were excluded if they did not return for re‐evaluation after reaching skeletal maturity or if they had bilateral femoral fractures.

### Re‐evaluation and imaging

For evaluation at skeletal maturity, each dog received physical and orthopaedic examinations by one of the authors, a health‐screen bloodwork panel (complete blood cell count and serum chemistry), a validated owner pain score evaluation [Canine Brief Pain Inventory (CBPI)] (Brown et al., 2008) and sedated radiographs. The sedation protocol included 0.2 to 0.3 mg/kg butorphanol combined with 4 to 6 mcg/kg dexmedetomidine administered either intravenously or intramuscularly depending upon patient disposition. Orthogonal radiographs, including a linear marker, of both femora were performed. The craniocaudal view was taken using a horizontal beam technique to decrease radiographic distortion, and a single, straight‐leg ventrodorsal view of the pelvis was also performed. Appropriate positioning of all radiographs was confirmed by one author (KLP) using criteria outlined by Dudley et al. (2006).

All radiographs, including those taken retrospectively for routine post‐operative follow‐up and those at skeletal maturity, were evaluated for evidence of femoral head malformation, closure of the greater trochanteric physis or a malformed greater trochanter. Fracture healing was graded *via* the International Society of Limb Salvage (ISLS) Grading Criteria (Glaser & Langlais, 1991) by two authors (LMM and KLP).

### Measurements

Each set of radiographs had specific measurements executed on the veterinary orthopaedic planning software (OrthoView; Materialise NV, Hampshire, United Kingdom). The anatomic lateral distal femoral angle (aLDFA), anatomic lateral proximal femoral angle (aLPFA), mechanical lateral distal femoral angle (mLDFA) and mechanical lateral proximal femoral angle (mLPFA) were all measured as described previously (Tomlinson et al., 2007) on the fractured femur post‐operatively, at each subsequent recheck and in both femora at the time of skeletal maturity. In addition, the anatomic caudal proximal femoral angle (aCdPFA), mechanical caudal distal femoral angle (mCdDFA), proximal and distal centres of rotation of normal angulation (CORONA1 and CORONA2, respectively) and angle of inclination (AOI) were measured as described previously on the affected femur post‐operatively, at each subsequent recheck and in both femora at skeletal maturity (Peterson et al., 2020; Rumph & Hathcock, 1990). In every radiograph, a circle of best fit over the femoral head was used to determine femoral head diameter. On the straight‐legged ventrodorsal pelvis view taken at skeletal maturity, the Norberg angle (NA) was measured at each coxofemoral joint as detailed previously (Gibbs, 1997).

**FIG 1:**
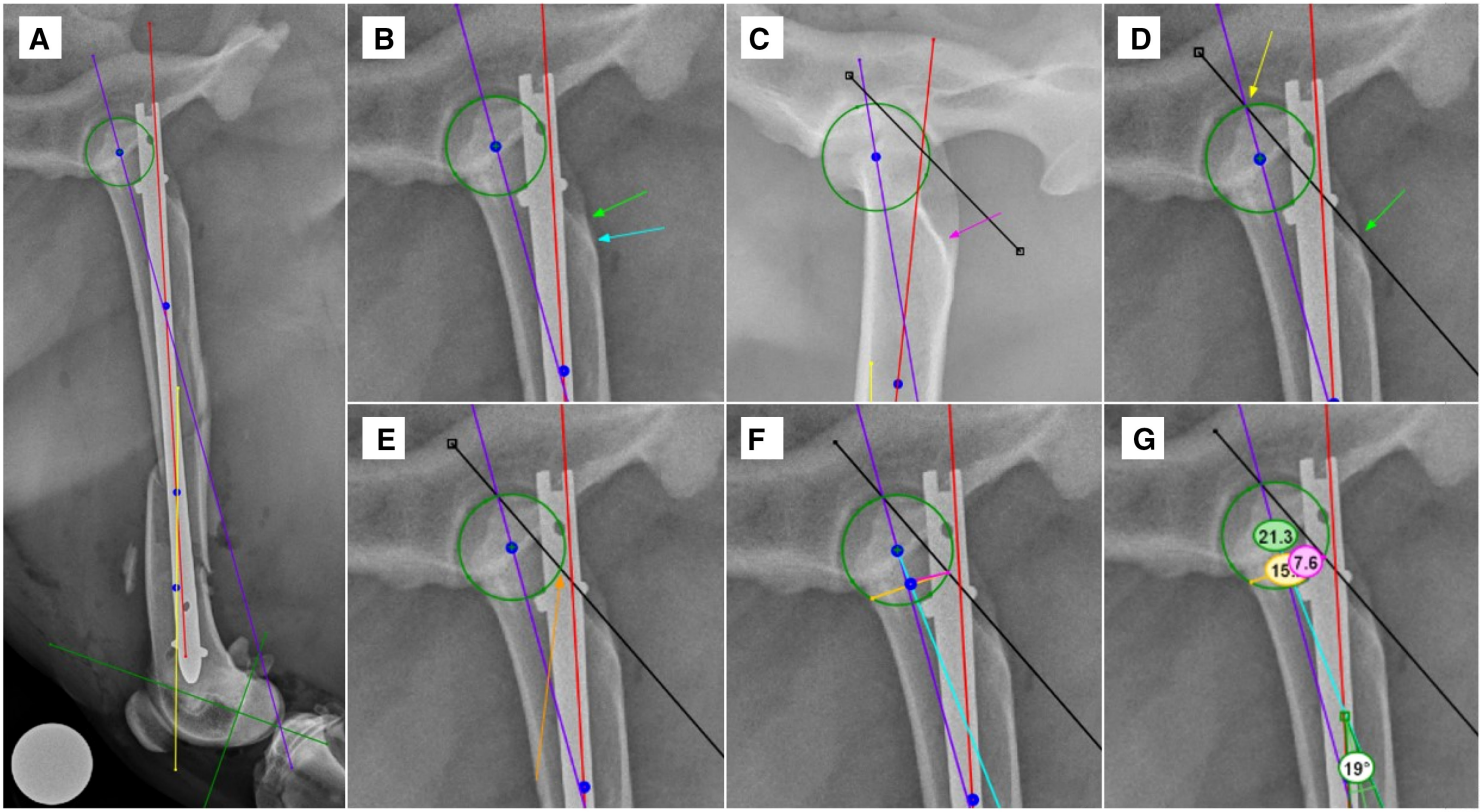
Procedural steps for the construction of Perrighetti line. (A) Orientation of all axes as described previously (Peterson et al., 2020), including circle of best fit over femoral head (green circle), proximal anatomic axis (red line) and mechanical axis (purple line). (B) Identification of the most caudal and proximal portion of the femoral neck (green arrow), likely to be part of the lesser trochanter in cases with less anteversion. The blue arrow is pointing to the most caudo‐distal aspect of the femoral neck. (C) In cases lacking caudal protrusion (common in highly anteverted femora), the reference point is placed at the most caudo‐proximal aspect of the femoral neck (pink arrow). (D) Construction of a Perrighetti line (black line) that intersects the spot (yellow arrow) where the mechanical axis crosses the proximal aspect of the circle of best fit over the femoral head, extending it to the tip of the caudal aspect of the femoral neck/lesser trochanter. (E) The intersection of the Perrighetti line with the most distal aspect of the circle of best fit of the femoral head (orange arrow) is where the neck would have contacted this area if not obscured by the nail. (F) Determination of the joint orientation line as described previously (Peterson et al., 2020). (G) Calculation of the aCdPFA by measuring the angle between the joint orientation line (blue line) and the proximal anatomical axis (red line).

Unfortunately, in 70% of cases, the AS‐ILN obscured the femoral neck as a landmark for the aCdPFA. To alleviate this, a line was created using consistently present anatomical landmarks that were not superimposed with the AS‐ILN to estimate the trajectory of the femoral neck. Multiple options were trialled using varying landmarks. The final methodology was selected based upon being the most accurate when compared to conventional aCdPFA angle measurement on the normal, contralateral femora included in this study. This novel “Perrighetti Line” estimates where the femoral neck would intersect the caudal aspect of the circle of best fit over the femoral head in cases where this landmark is obscured by the AS‐ILN. Step‐by‐step directions are detailed in Fig 1. To ensure consistency between patients, this method was used in addition to the previously described method (Peterson et al., 2020) to determine the aCdPFA in every case.

All measurements were separately performed by two authors (LMM and KLP) and were assessed for interobserver variability. The mean of the two measurements was then calculated and used for statistical analysis.

### Statistical analysis

Interobserver variability of each measurement variable was determined using an intraclass correlation coefficient (ICC). Due to the small sample size, an assumption that data were not normally distributed was made. A Wilcoxon signed rank test was used to assess the femoral measurements for significant differences, using the unaffected femur as a control in each dog at skeletal maturity. In addition, the Wilcoxon signed rank test was used to evaluate whether there were any significant changes to femoral angles of the fractured femur noted immediately preoperatively to those at skeletal maturity. A value of *P* < .05 was considered significant.

## RESULTS

### Case population

Thirteen skeletally immature dogs sustained femoral diaphyseal fractures stabilised with an AS‐ILN. Two had died at the time of correspondence, unrelated to the femoral fracture, but one was included within the study as skeletal maturity had been reached by the final recheck. One owner was unable to be contacted, and one owner declined participation. Ten dogs were included. Breeds included Labrador retriever (*n* = 4), Labradoodle (*n* = 2), Brittany spaniel (*n* = 1), Bernese Mountain dog (*n* = 1) and mixed breed (*n* = 2). There were five entire males, four entire females and one neutered male. Fractures were caused by vehicular trauma (*n* = 6), jumping out of a moving car (*n* = 2), landing abnormally after jumping (*n* = 1) and running into a wall (*n* = 1). Two dogs fractured the left femur and eight the right. Fracture configuration consisted of four simple (three transverse and one short oblique) and six comminuted fractures.

All AS‐ILN were placed *via* open reduction and internal fixation with normograde placement from proximally. Bolt configuration was 1:1 (*n* = 6), 1:2 (*n* = 2) or 2:2 (*n* = 2). Mean age at the time of fracture repair was 5.9 months (range 4 to 7 months). Mean age at re‐evaluation was 3.1 years (range 1 to 5.5 years).

### Canine Brief Pain Inventory results

Nine dogs had a CBPI performed; all dogs had a very good (*n* = 2) or excellent (*n* = 7) quality of life. As noted previously, one dog had died at the time the study was performed, but as it had been re‐evaluated after skeletal maturity, it was included; however, this dog (dog 1) was unable to contribute to the CBPI data. Six of the nine dogs scored a 0 (out of a total of 100 points), indicating a lack of appreciable pain and no disruption in clinical orthopaedic function. Three dogs were reported to have minimal scores (range from 7 to 10), indicating minor impairment in function and/or subtle pain. Results from each CBPI are summarised in Table 1.

**Table 1:** Canine Brief Pain Inventory results

Patient	Description of pain (0 to 40 scale)	Description of function (0 to 60 scale)	Overall impression
2	0	0	Excellent
3	0	0	Excellent
4	0	0	Excellent
5	0	0	Excellent
6	0	0	Excellent
7	0	0	Excellent
8	3	4	Very good
9	3	7	Very good
10	3	5	Excellent

Scale: 0 = no functional impairment or pain, 40 = severe pain and 60 = severe impairment

### Orthopaedic examination evaluation and bloodwork analysis

At skeletal maturity, one dog manifested lameness. Lameness scores and relevant orthopaedic examination findings are summarised in Table 2. Each dog, of nine where bloodwork was performed, had at least one value out with reference range; however, none were considered clinically significant. Each dog was deemed healthy to undergo sedation.

**Table 2:** Orthopaedic examination findings at skeletal maturity

Patient	Lameness?	Abnormalities
1	None	Pain on full extension and abduction of both coxofemoral joints and with tail jack
2	None	Pain on full extension and abduction of both coxofemoral joints and with tail jack
3	None	None
4	None	None
5	None	None
6	None	Pain response with tail jack and lumbosacral palpation (pain with full extension of coxofemoral joints bilaterally, but suspect referred spinal pain); pain response with full extension of elbows and palpation of medial coronoid bilaterally; pain with full extension of the shoulders and with biceps tendon test; pain with palpation of iliopsoas bilaterally; left medial patellar subluxation
7	None	Bilateral bidirectional patellar subluxation
8	None	Mildly decreased abduction of the right coxofemoral joint; slightly increased resistance to abduction of the right coxofemoral joint; mild–moderate muscle atrophy right pelvic limb
9	Yes – right pelvic limb – grade III/V	Bilateral pain with elbow extension, pelvic limb muscle atrophy (right > left), right tibial thrust/cranial drawer, joint effusion, buttress, pain with full stifle extension; pain on extension/abduction of both hips
10	None	Pain response upon palpation over distal aspect of radial bone plate

### Radiographic evaluation

Radiographic findings are reported in Table 3. Eight of the ten dogs had undergone recheck fracture evaluations prior to skeletal maturity: five with one recheck and two with two rechecks. No case was noted to have evidence of PFM, coxofemoral subluxation or premature closure of femoral physes.

**Table 3:** Radiographic findings

Patient	ISLS grade			Malformed femoral head			Greater trochanter physis closure			Malformed greater trochanter			Other radiographic pathology at SM
Age at time of fracture (m)	Recheck			Recheck/age at recheck			Recheck/age at recheck			Recheck/age at recheck			
	1	2	SM	1	2	SM	1	2	SM	1	2	SM	
1 (7 m)	III	III	IV	No (8 m)	No (8.5 m)	No (1 yr)	No (8 m)	No (8.5 m)	Yes (1 yr)	No (8 m)	No (8.5 m)	No (1 yr)	
2 (6 m)	IV		IV	No (8 m)		Yes^§^ (5.5 yr)	Yes* (8 m)		Yes (5.5 yr)	No (8 m)		No (5.5 yr)	Bilateral coxofemoral degenerative changes
3 (7 m)	I	IV	IV	No (8 m)	No (9 m)	No (5 yr)	–^‡^ (8 m)	–^‡^ (9 m)	Yes (5 yr)	No (8 m)	No (9 m)	No (5 yr)	
4 (6 m)	IV		IV	No (9 m)		No (3.5y)	Yes* (9 m)		Yes (3.5 yr)	No (9 m)		No (3.5 yr)	
5 (7 m)			IV			No (1.5 yr)			Yes (1.5 yr)			No (1.5 yr)	Enthesophyte at right (surgical) greater trochanter
6 (4 m)			IV			No (1 yr)			Yes (1 yr)			No (1 yr)	
7 (6.5 m)	I		IV	No (7 m)		No (1 yr)	No (7 m)		Yes (1 yr)	No (7 m)		No (1 yr)	Bilateral enthesophytes at greater trochanter
8 (4 m)	IV		IV	No (5 m)		No (4 yr)	No (5 m)		Yes (4 yr)	No (5 m)		No (4 yr)	Enthesophyte at right (surgical) greater trochanter
9 (6 m)	III	IV	IV	No (7 m)	No (7.5 m)	Yes (4 yr)	No (7 m)	No (7.5 m)	Yes (4 yr)	No (7 m)	No (7.5 m)	No (4 yr)	Sclerosis of right (surgical) greater trochanter. Enthesophyte at left greater trochanter. Bilateral coxofemoral degenerative changes
10 (5 m)	IV		IV	No (7 m)		No (4.5 yr)	Yes* (7 m)		Yes (4.5 yr)	No (7 m)		No (4.5 yr)	Sclerosis of right (surgical) greater trochanter

ISLS International Society of Limb Salvage grade, m Months, SM Skeletal maturity, yr Years

* Normal time for physeal closure (≥7 months)

^‡^ Bolt overriding physis

^§^ Bilaterally affected

**FIG 2:**
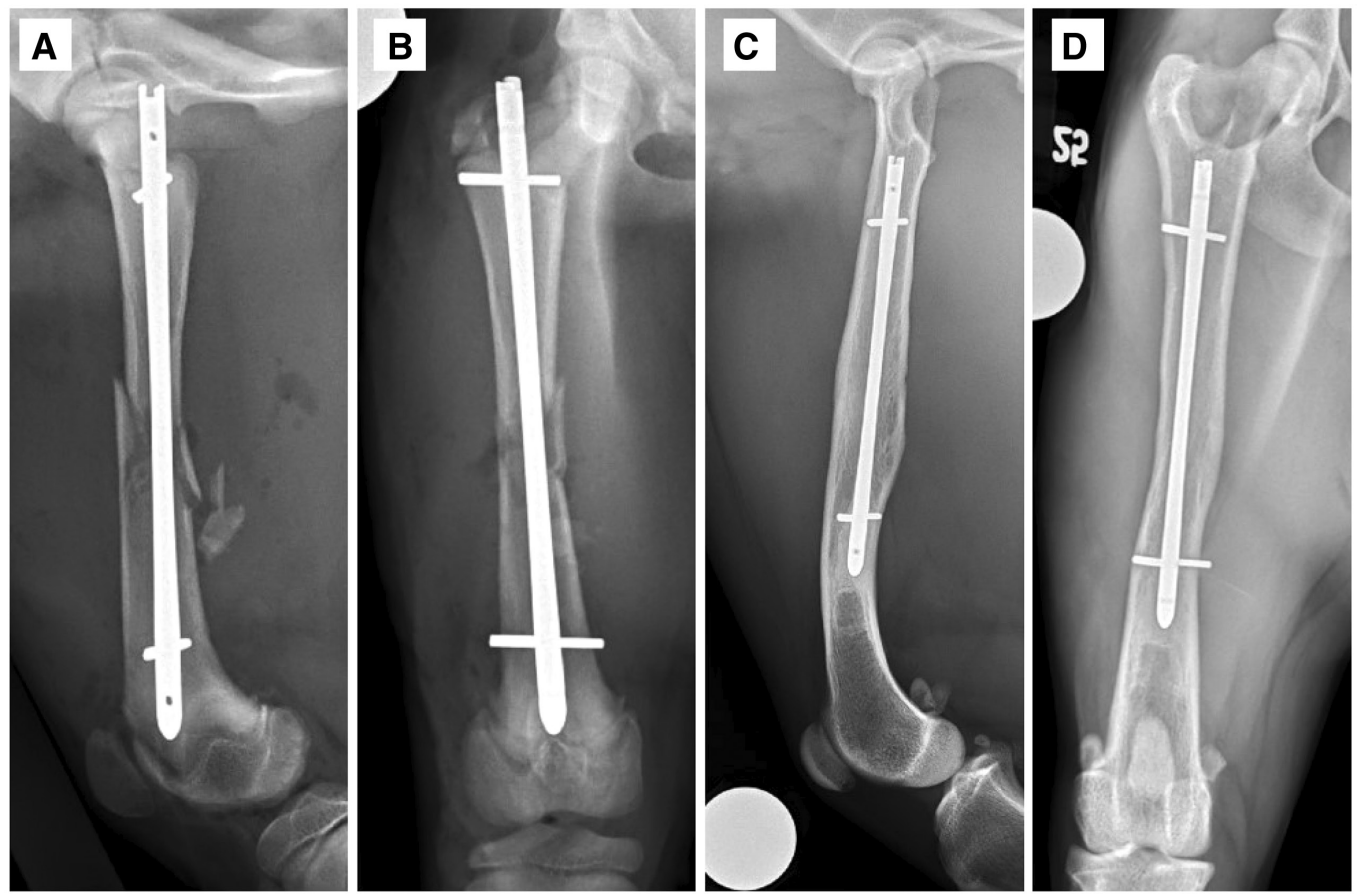
Lateral and craniocaudal views of the left femur immediately post‐operatively (A, B) and at skeletal maturity (C, D) from dog 6 (A, B) show a mid‐diaphyseal comminuted fracture of the left femur stabilised using a 4‐ × 102‐mm AS‐ILN in a 1:1 bolt configuration. Note the absence of any PFM at skeletal maturity (C, D).

**FIG 3:**
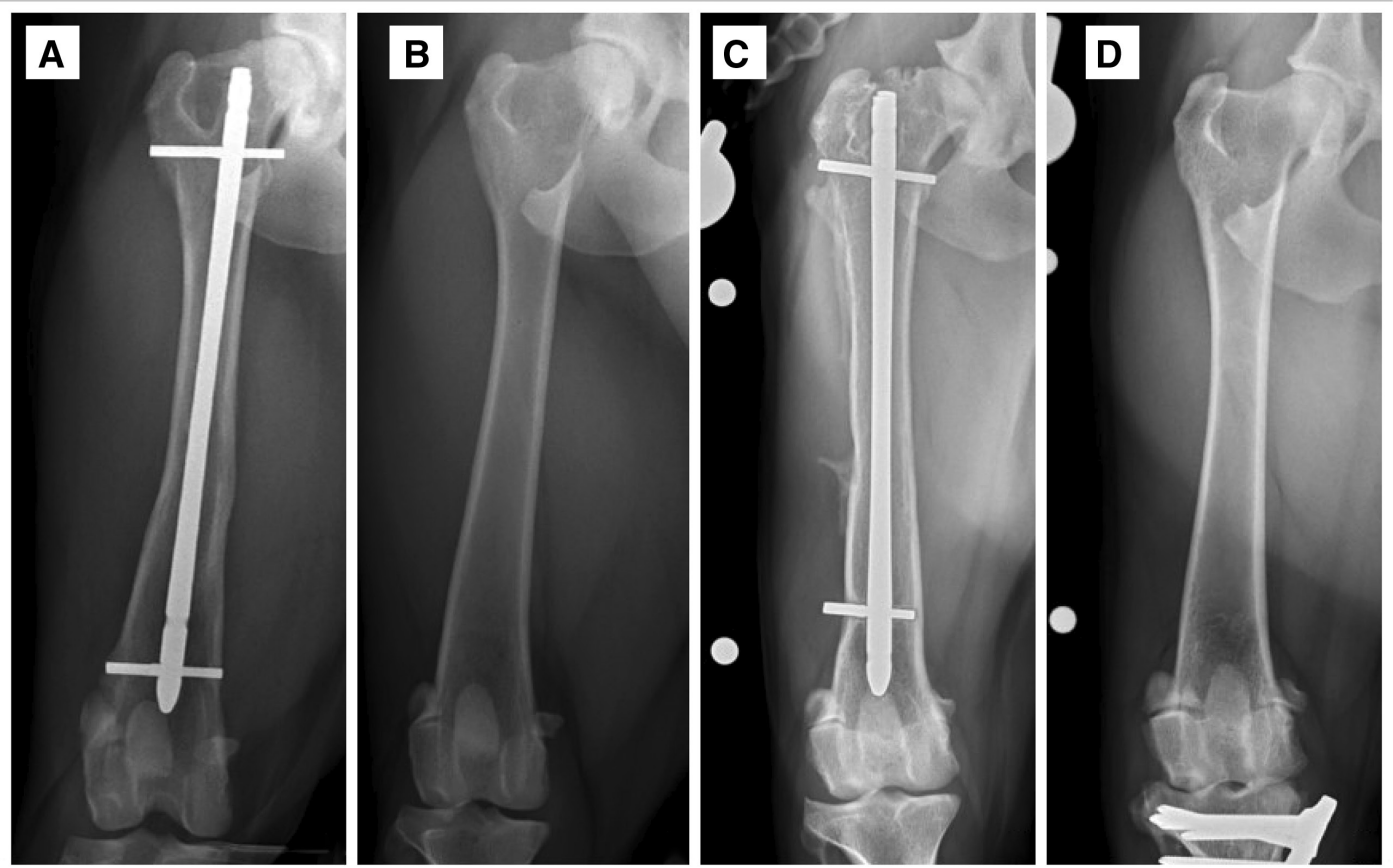
Craniocaudal views of the right femur from dogs 2 (A, B) and 9 (C, D) taken at skeletal maturity. (A) A 6 × 172 mm AS‐ILN in a 1:1 bolt configuration is present. Bony union and re‐corticalisation have been achieved. Moderate femoral neck thickening and femoral head remodelling are appreciated. Mild osteophysis is observed at the cranial pole of the acetabulum. (B) Similar degenerative changes are observed in the left coxofemoral joint of dog 2. (C) Bony union of a previous proximal diaphyseal fracture of the right femur stabilised using a 7 × 172 mm AS‐ILN in 1:1 bolt configuration. Severe changes to the right coxofemoral joint include femoral neck thickening, femoral head remodelling and flattening, and osteophytosis of the cranial pole of the acetabulum. There is sclerosis of the greater trochanter. Periarticular osteophytosis is also observed at the level of the stifle. (D) Moderate degenerative changes are observed at the left coxofemoral joint, including thickening of the femoral neck with an associated Morgan's line and osteophytosis of the cranial pole of the acetabulum. An enthesophyte is present just proximal to the greater trochanter. Severe periarticular osteophytosis is evident affecting the stifle joint. The head of a previously placed TPLO bone plate is present with associated locking screws.

**FIG 4:**
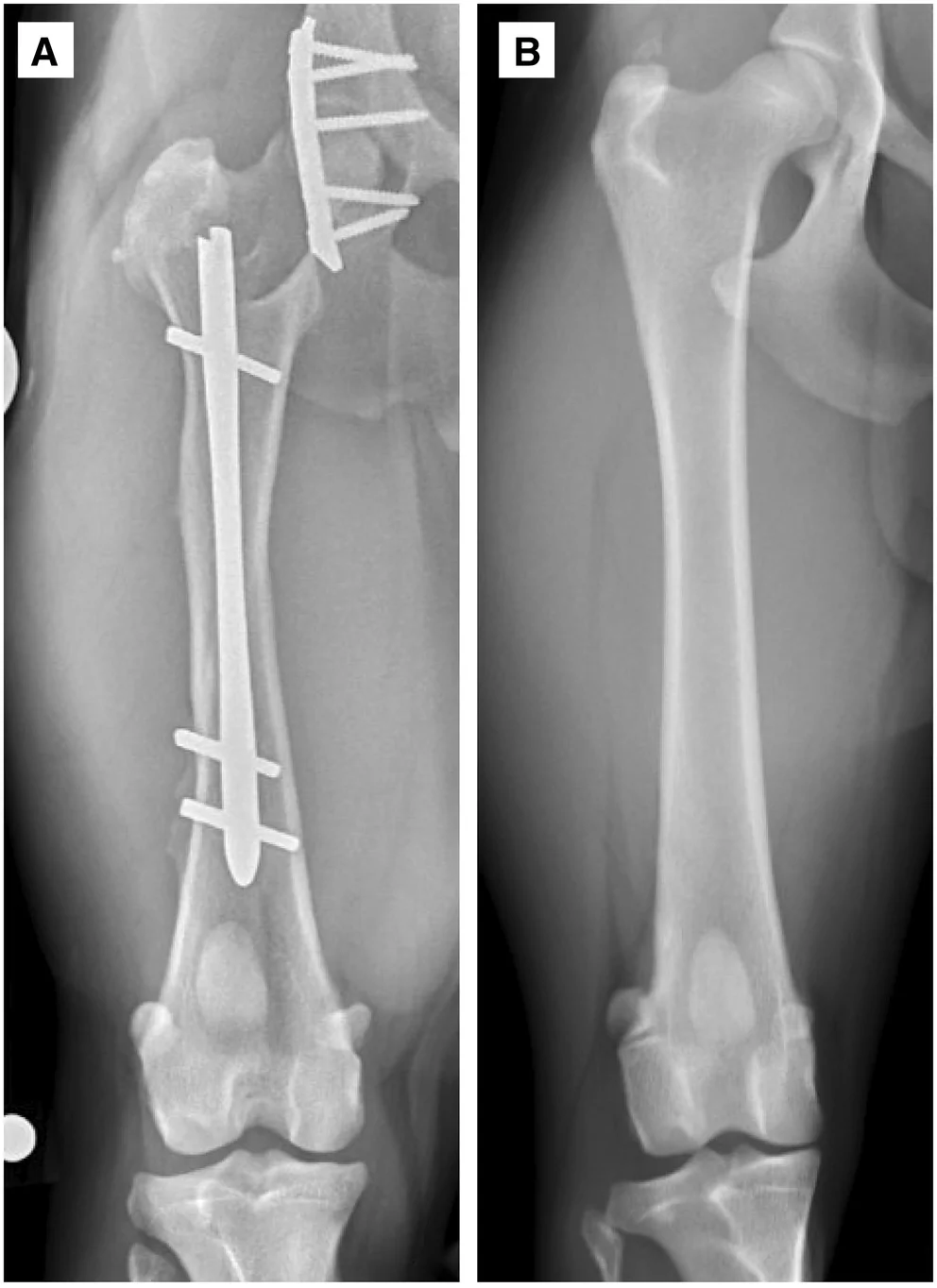
(A) Craniocaudal view of the right femur at skeletal maturity for dog 8 showing a small enthesophyte at the distolateral aspect of the greater trochanter. Multiple implants are present, including a 6 × 122 mm AS‐ILN in 1:2 bolt configuration as well as a 2.0‐mm acetabular bone plate. No evidence of degenerative change is observed within the coxofemoral joint. (B) Dog 7 showing an enthesophyte just proximal to the greater trochanter of the left, non‐surgical femur at skeletal maturity.

On radiographic evaluation at skeletal maturity, four of the ten dogs had no radiographic evidence of abnormalities at the proximal femur (Fig 2C, D). Of the six dogs with radiographic pathology, two dogs had bilateral degenerative changes to the coxofemoral joints, including thickened femoral necks, remodelling of the femoral head and shallow acetabulae; pathologic changes were equivalent between sides on one dog, but differences were appreciated for the other case with the surgical limb having more advanced disease (Fig 3A–D, respectively). Four dogs had enthesophytes at the greater trochanter [one bilateral, two affecting the surgical limb and one affecting the non‐surgical limb (Fig 4B)]. Two dogs had sclerosis of the greater trochanter on the surgical limb.

### Radiographic measurements

The comparison between CORONA1, CORONA2, aCdPFA, mCdDFA, aLDFA, mLDFA, aLPFA, mLPFA, AOI, femoral head diameter and NA measurements between the surgical and normal limbs at skeletal maturity, as well as the surgical limb immediately post‐operatively compared with the surgical limb at skeletal maturity are detailed in Table 4. Only the CORONA1 value between the surgical limb and non‐surgical limb at skeletal maturity was noted to be statistically significantly different (*P* = .041).

**Table 4:** Results of Wilcoxon signed rank tests for femoral measurements

Measurement	Value surgical limb *versus* normal limb at skeletal maturity	*P*‐value surgical limb immediately post‐operatively and at skeletal maturity
CORONA 1	0.04129037199*	0.1735339885
CORONA 2	0.5699760828	0.09196645248
aCdPFA	0.8633720617	0.3123642584
mCdDFA	0.07186843871	0.09196645248
aLDFA	0.162099634	0.5887439796
mLDFA	0.8633720617	0.6573376847
aLPFA	0.4973936088	0.6573376847
mLPFA	0.4001854054	0.5559491479
AOI	0.7314717145	0.8429988116
Femoral head diameter	0.908616128	0.06623128469
NA	0.3174382746	N/A

* Statistically significant (*P* < .05)

### Intraclass correlation

Each measurement variable and associated ICC are summarised in Table 5. Overall, interobserver reliability was good to excellent in 90% of the measured variables. CORONA 2 had moderate reliability. No measurement had poor reliability.

**Table 5:** Intraclass correlation coefficients for femoral measurements

Measurement	ICC	Reliability result
CORONA 1	0.7775997259	Good
CORONA 2	0.5071693471	Moderate
aCdPFA	0.949726326	Excellent
mCdDFA	0.8295478971	Good
aLDFA	0.8560096473	Good
mLDFA	0.8865193825	Good
aLPFA	0.9494492089	Excellent
mLPFA	0.9513974707	Excellent
AOI	0.8915782005	Good
Femoral head diameter	0.8669867091	Good
NA	0.8714149414	Good

## DISCUSSION

In this skeletally immature population, AS‐ILN use to stabilise diaphyseal femoral fractures was not associated with PFM. While direct comparison between studies is not appropriate, these results show a stark contrast to the previous study using intramedullary pins in a similar patient cohort, where 87% of cases developed malformations (Black & Withrow, 1979).

The results of owner perceived quality of life within this cohort were excellent (77.8%) or very good (22.2%) in all cases. One dog (dog 10), noted to have a score of 8, was a previous polytrauma patient; at the time of re‐evaluation, chronic implant‐associated infection from implants used to stabilise a concomitant radius and ulna fracture was noted. The presence of mild pain and suboptimal function is considered likely to be due to this as the right thoracic limb was the only area where pain was elicited during examination. Orthopaedic examinations on the other two dogs that had CBPI scores above zero showed evidence of additional orthopaedic ailments, including a right cranial cruciate ligament rupture (dog 9) and decreased range of motion of the hip without evidence of pain (dog 8). Dog 8 sustained multiple fractures in addition to the femoral fracture, including ipsilateral acetabular and tibial fractures. The plate used to stabilise the acetabular fracture may have impinged upon range of motion. While highly likely that there was another orthopaedic reason behind the minimally decreased function and/or subtle pain in these two dogs, it was not possible to definitively rule out a consequence of AS‐ILN‐use as a contributor.

On orthopaedic examinations performed at skeletal maturity, only one dog (dog 9) had evidence of lameness; the lameness was associated with the right pelvic limb, where the femur had fractured previously. However, this dog was also noted to have significant right stifle instability as well as pain upon full stifle extension. While dog 9 was also witnessed to have pain associated with extension and abduction of the right coxofemoral joint, clinical lameness is more often associated with the stifle than the coxofemoral joint in cases where pain is appreciated to be affecting both joints. It is considered probable that this dog's lameness was, at least predominantly, associated with the stifle rather than the coxofemoral joint; however, this cannot be definitively proven.

Only three of the ten dogs had completely unremarkable orthopaedic examinations. Four cases had pain associated with extension and abduction of the coxofemoral joints bilaterally, and in all but one of these cases, the pain associated with manipulation was equivalent between the two sides. In addition, in three of the four cases where pain was associated with the coxofemoral joints (dogs 1, 2 and 6), dogs were also noted to have signs consistent with spinal pain; as such, a contribution from referred spinal pain cannot be excluded. Only two of the four dogs with coxofemoral joint pain were appreciated to have radiographic pathology within these joints, and they were both bilaterally affected.

Given the bilaterally symmetrical pain responses upon extension and abduction of the coxofemoral joints, it was felt more likely that these were due to pathology unrelated to the original femoral fracture, with hip dysplasia and associated osteoarthritis being the most likely differential. Radiographic findings seemed to support this, with degenerative changes to the coxofemoral joints being noted bilaterally in two dogs. In one case, these changes were of comparable radiographic severity bilaterally (Fig 3A, B). In the one dog in which more severe degenerative changes were appreciated on the surgical limb (Fig 3C, D), no evidence of the pathologies reported previously (Black & Withrow, 1979) was noted during follow‐up prior to skeletal maturity. The previous study noted that proximal femoral changes were able to be appreciated within 2 to 6 weeks following surgical stabilisation of femoral fractures with intramedullary implants (Black & Withrow, 1979); in our dog with asymmetric degeneration observed only at skeletal maturity, follow‐up radiographs were available at 6 weeks post‐operatively showing no radiographic evidence of malformation. In addition, the changes at skeletal maturity (thickened femoral neck, large and flattened femoral head) directly contrast with those cited previously (Black & Withrow, 1979). The authors therefore consider it more likely that these changes are reflective of hip dysplasia and associated osteoarthritis, more severe on the operated side than the contralateral.

Whether post‐traumatic osteoarthritis could play a role in the more severe radiographic signs noted on one side remains speculative. Daly (1978) reported that degeneration of the coxofemoral joints tended to occur secondary to trauma sustained at the time of femoral neck fracture, including damage to the joint capsule and muscle, which caused loss of joint stability and resulted in more severe changes in the traumatised joint relative to the unaffected. The dog with asymmetric coxofemoral joint changes was hit by a car, qualifying as high‐energy trauma, and this could certainly have destabilised an already dysplastic joint. While this remains postulative, based upon the previous publication (Black & Withrow, 1979) the changes do not appear to reflect those expected to occur secondary to placement of the intramedullary implant itself.

In the eight dogs that had radiographic re‐evaluation prior to skeletal maturity, none were noted to have evidence of coxa valga, PFM, coxofemoral subluxation, malformed femoral head or premature closure of the greater trochanteric physis. While there is a range over which this physis can close (Sutton et al., 2018), the earliest closure that has been reported is at 7 months. In all cases included in this study, the greater trochanteric physis was radiographically open at the time of surgery and all but one of these cases were re‐evaluated when they reached at least 7 months of age. The greater trochanteric physis remained open in six out of seven cases with short‐term radiographic follow‐up which indicates that growth potential remained in these dogs and hereby that malformation had the potential to develop, although the degree of remaining growth and deformity potential clearly remains unknown. In the one dog (dog 8) in which the final post‐operative recheck was performed at 5 months of age, upon re‐evaluation at 4 years of age, the only abnormal radiographic finding was the presence of an enthesophyte at the greater trochanter on the previously operated side; there was no evidence of degenerative disease or PFM (Fig 4A).

**FIG 5:**
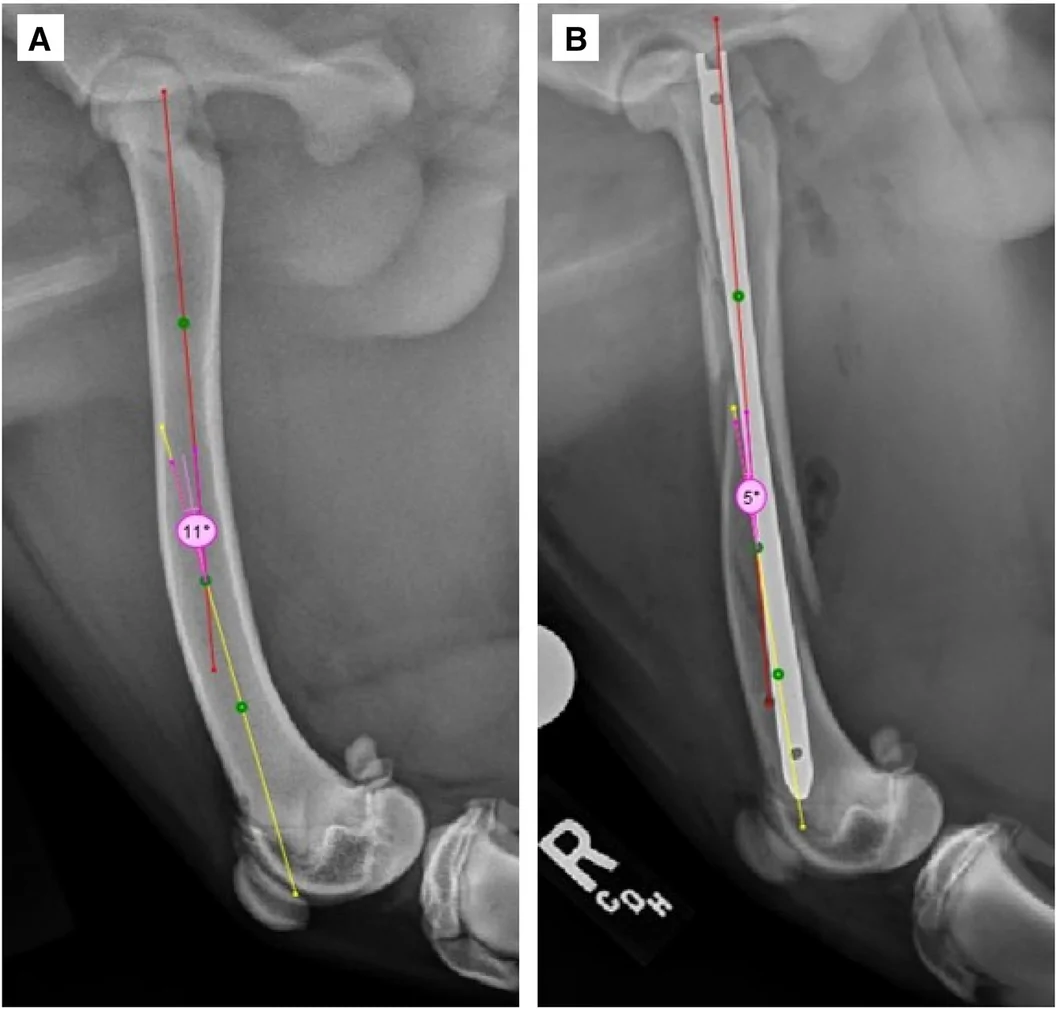
Mediolateral views of the unaffected femur taken preoperatively (A) and the affected femur (B) taken post‐operatively. The red line represents the proximal anatomic axis. The yellow line represents the intermediate anatomic axis. The angle outlined in pink is the CORONA 1 measurement. Note the reduction in CORONA 1 which manifests post‐operatively as a result of over‐reduction of the fracture, which is necessary in order to plate a straight intramedullary implant into a bone with natural procurvatum.

CORONA 1 was the only measurement where a significant difference was found between groups, specifically comparing the unaffected femur to the surgical femur at the time of skeletal maturity. As there was no significant difference in the CORONA 1 measurement between the affected femur immediately post‐operatively and at skeletal maturity, this implies that the difference in measurement between the operated and nonoperated femora is a direct result of the surgical reduction rather than a difference in growth following stabilisation (Fig 5). This alteration quantifies a relative recurvatum which is anticipated when using a straight intramedullary implant to stabilise a bone with a natural procurvatum. While individual fractures resulted in different magnitudes of relative recurvatum, likely dictated by the level at which the fracture occurred, the initial degree of procurvatum and the degree of comminution, the change in angulation of the femur did not have any notable clinical significance. Similarly, clinical consequences of reducing natural femoral procurvatum have not been reported in adult dogs undergoing fracture stabilisation using interlocking nails previously. Having said this, in cases where over‐reduction is considered undesirable, such as where anatomic reconstruction is preferred, this could be avoided *via* the placement of a commercially available precountered nail (Roels et al., 2024) or a straight nail bent intraoperatively to match the anatomic procurvatum of the individual case (Perry & Wesslen, 2025).

Enthesophytes at the greater trochanter were noted in four cases but not consistently affecting the surgically treated limb. In two of the four cases, enthesophytes were only noted on the ipsilateral limb. However, in one case they were noted bilaterally, and in one case only affecting the contralateral femur. It is tempting to ascribe the enthesophyte formation to the surgical approach, with retraction or penetration of the gluteal musculature occurring during preparation for and placement of the AS‐ILN, but this explanation cannot clearly be extrapolated to all cases. In the one case with enthesophyte formation only affecting the non‐surgical limb, coxofemoral osteoarthritis likely secondary to dysplasia was radiographically confirmed and the enthesophyte may represent a part of that same pathology. However, for the bilaterally affected case, no other orthopaedic conditions were diagnosed upon clinical or radiographic examination and, as such, the cause for enthesophyte formation remains unknown.

While comparing results between studies is always fraught with complications, it is interesting to consider potential reasons for the contrasting results between the previous study (Black & Withrow, 1979) and this current one. In three of the eight dogs included within the previous study cautioning against intramedullary implant use (Black & Withrow, 1979), intramedullary pinning was used as the sole stabiliser. As far back as 1976 (Braden & Brinker, 1976), use of an intramedullary pin in isolation was shown to be insufficient for fracture management, due to the inability to effectively neutralise rotational and compressive forces (Beale, 2004; Dallman et al., 1990). While not explicitly related to juvenile patients, pin migration has been reported as a complication associated with intramedullary pin use, with or without concurrent use of cerclage wires (Hunt et al., 1980; Libardoni et al., 2018; Phillips, 1979; Raouf et al., 2019; Talaat et al., 2022; Vaughan, 1975). Without the ability to immobilise the pin, it is possible that minor motion may contribute to damage within the medullary cavity and potentially disturb the epiphyseal blood supply, which may have contributed to the malformations reported previously in juvenile patients (Black & Withrow, 1979).

Another potential contributor to PFM in the previous study (Black & Withrow, 1979) was the practice of retrograde IM pin placement which is well recognised to compromise proximal IM pin positioning and may be more likely to result in altered development. Indeed, in one case within that study, pin penetration of the greater trochanter was reported in a case that subsequently developed malformation of the trochanter. In the current study, all femurs were prepared for the AS‐ILN in a normograde fashion with resultant predictable and consistent proximal pin positioning which could reasonably be anticipated to reduce risks of PFM. In children, even the use of normograde approaches through either the piriformis fossa or the greater trochanter has been associated with avascular necrosis of the femoral head and the development of deformity (Buford et al., 1998; González‐Herranz et al., 1995) and as such, alternative approaches have been investigated, including using the lateral aspect of the greater trochanter as a starting point (Keeler et al., 2009), or the use of more flexible interlocking nails (Jencikova‐Celerin et al., 2008). While the results of this study do not indicate that such considerations are necessary based upon this small case population, they remain possibilities for consideration when attempting to preserve the tenuous vascularity in this anatomic region.

With an AS‐ILN, slack is eliminated whilst allowing effective neutralisation of bending, torsion and compressive forces (Lansdowne et al., 2007). It is possible that the enhanced stability of an AS‐ILN, when compared with an intramedullary pin, is at least partially responsible for the absence of PFM in this report. As such, the results of this study cannot be extrapolated to include other intramedullary implants which allow continued instability. In addition, over time surgical techniques have improved to minimise trauma to the soft tissue envelope. The transition away from anatomic reduction and rigid stabilisation and towards open but do not touch approaches may also have contributed to the positive results of this study by prioritising preservation of the lateral circumflex femoral, medial circumflex femoral and caudal gluteal arteries, which are known to provide the major blood supply to the femoral head and greater trochanter (Bassett et al., 1969).

There are several limitations to this study. As intramedullary fixation techniques were previously considered relatively contraindicated in juvenile patients (Black & Withrow, 1979), stabilisation with an AS‐ILN was not performed commonly and therefore the number of cases is small. The Wilcoxon signed rank test was chosen specifically with this in mind, being appropriate for small data sets with non‐normally distributed data; however, the risk for a type II error must still be considered. While the contrast between the previous study (Black & Withrow, 1979) and this seems convincing, it is possible that PFM may still occur following use of an AS‐ILN in skeletally immature dogs, and that the risk of this is simply reduced. A prospective study design with larger case numbers would be necessary to elucidate this, as well as to investigate potential risk factors for PFM, and is planned in the future. Due to the retrospective nature of the study, perioperative protocols were not standardised. Not all dogs returned for follow‐up in the immediate post‐operative period, so time to clinical union and premature closure of the proximal femoral physes was not always able to be evaluated. In addition, perioperative radiographs, including those during fracture rechecks, did not undergo the same scrutiny as those for final re‐evaluation at skeletal maturity. The presence of callus also made some measurements difficult to attain due to landmarks being obscured, and this likely explains why measurements relying upon identification of cortical margins for diaphyseal measurements, namely CORONA 1 and CORONA 2, had the lowest ICC; notably, this is the first time that use of these measurements has been reported in fracture patients. The landmarks required for measurement of the aCdPFA were obstructed by the AS‐ILN in most cases, necessitating the creation of the Perrighetti line. Use of this line to measure the aCdPFA had 94.8% accuracy when performed on the normal femur in comparison with the conventionally measured aCdPFA (Peterson et al., 2020); however, as it is a novel technique, it has not been validated.

Based upon this case population, the use of an AS‐ILN to stabilise femoral diaphyseal fractures in skeletally immature dogs can be performed without significant risk of clinical dysfunction or development of PFM using current standard surgical practices. A combination of effects including a more biologic approach to fracture repair and use of an AS‐ILN to create a more suitable environment for fracture healing may contribute to the outcomes reported in this patient population.

### Author contributions

L. M. Meneghetti analysed data, evaluated clinical cases and radiographs, and wrote the manuscript. S. R. O'Neill wrote the initial grant for the study and identified medical records. K. L. Perry designed the study, evaluated cases and radiographs, and edited the manuscript.

### Conflict of interest

The authors have no conflicts of interest to declare. Dr Perry is an Associate Editor of the Journal of Small Animal Practice and co‐author of this article. She was excluded from editorial decision‐making related to the acceptance of this article for publication.

### Funding

Funding was provided for this study from the College of Veterinary Medicine's Endowed Research Funds, specifically from Babe's Fund for Canine Research.

## Data Availability

The data that support the findings of this study are available from the corresponding author upon reasonable request.

## References

[jsap70129-bib-0001] Aithal, H.P. , Singh, G.R. & Bisht, G.S. (1999) Fractures in dogs: a survey of 402 cases. Indian Journal of Veterinary Surgery, 20, 15–21.

[jsap70129-bib-0002] Bassett, F.H., 3rd , Wilson, J.W. , Allen, B.L., Jr. & Azuma, H. (1969) Normal vascular anatomy of the head of the femur in puppies with emphasis on the inferior retinacular vessels. Journal of Bone and Joint Surgery, American Volume, 51, 1139–1153. 5817168

[jsap70129-bib-0003] Beale, B. (2004) Orthopedic clinical techniques femur fracture repair. Clinical Techniques in Small Animal Practice, 19, 134–150. 15712460 10.1053/j.ctsap.2004.09.006

[jsap70129-bib-0004] Black, A.P. & Withrow, S.J. (1979) Changes in the proximal femur and coxofemoral joint following intramedullary pinning of diaphyseal fractures in young dogs. Veterinary Surgery, 8, 19–24.

[jsap70129-bib-0005] Braden, T.D. & Brinker, W.O. (1976) Radiologic and gross anatomic evaluation of bone healing in the dog. Journal of the American Veterinary Medical Association, 169, 1318–1323. 1002599

[jsap70129-bib-0006] Braden, T.D. , Eicker, S.W. , Abdinoor, D. & Prieur, W.D. (1995) Characteristics of 1000 femur fractures in the dog and cat. Veterinary and Comparative Orthopaedics and Traumatology, 8, 203–209.

[jsap70129-bib-0007] Brown, D.C. , Boston, R.C. , Coyne, J.C. & Farrar, J.T. (2008) Ability of the Canine Brief Pain Inventory to detect response to treatment in dogs with osteoarthritis. Journal of the American Veterinary Medical Association, 233, 1278–1283. 19180716 10.2460/javma.233.8.1278 PMC2896492

[jsap70129-bib-0008] Buford, D., Jr. , Christensen, K. & Weatherall, P. (1998) Intramedullary nailing of femoral fractures in adolescents. Clinical Orthopaedics, 350, 85–89. 9602805

[jsap70129-bib-0009] Cabassu, J.‐P. (2001) Elastic plate osteosynthesis of femoral shaft fractures in young dogs. Veterinary and Comparative Orthopaedics and Traumatology, 14, 40–45.

[jsap70129-bib-0010] Chapman, J.R. , Harrington, R.M. , Lee, K.M. , Anderson, P.A. , Tencer, A.F. & Kowalski, D. (1996) Factors affecting the pullout strength of cancellous bone screws. Journal of Biomechanical Engineering, 118, 391–398. 8872262 10.1115/1.2796022

[jsap70129-bib-0011] Dallman, M.J. , Martin, R.A. , Self, B.P. & Grant, J.W. (1990) Rotational strength of double‐pinning techniques in repair of transverse fractures in femurs of dogs. American Journal of Veterinary Research, 51, 123–127. 2301811

[jsap70129-bib-0012] Daly, W.R. (1978) Femoral head and neck fractures in the dog and cat: a review of 115 cases. Veterinary Surgery, 7, 29–38.

[jsap70129-bib-0013] Dudley, R.M. , Kowaleski, M.P. , Drost, W.T. & Dyce, J. (2006) Radiographic and computed tomographic determination of femoral varus and torsion in the dog. Veterinary Radiology & Ultrasound, 47, 546–552. 17153063 10.1111/j.1740-8261.2006.00184.x

[jsap70129-bib-0014] Gibbs, C. (1997) The BVA/KC scoring scheme for control of hip dysplasia: interpretation of criteria. The Veterinary Record, 141, 275–284. 9316245

[jsap70129-bib-0015] Glaser, D. & Langlais, F. (1991) The ISOLS radiological implants evaluation system. In: Langlais, F. & Tomeno, B. (Eds.) Limb salvage: major reconstructions in oncologic and nontumoral conditions. Berlin: Springer, pp. xxiii–xxxi.

[jsap70129-bib-0016] González‐Herranz, P. , Burgos‐Flores, J. , Rapariz, J.M. , Lopez‐Mondejar, J.A. , Ocete, J.G. & Amaya, S. (1995) Intramedullary nailing of the femur in children. Effects on its proximal end. Journal of Bone and Joint Surgery, British Volume, 77, 262–266. 7706343

[jsap70129-bib-0017] Herzog, B. (1977) End results of intramedullary nailing of femoral fractures in children. Journal of Trauma and Acute Care Surgery, 17, 174.

[jsap70129-bib-0018] Hunt, J.M. , Aitken, M.L. , Denny, H.R. & Gibbs, C. (1980) The complications of diaphyseal fractures in dogs: a review of 100 cases. The Journal of Small Animal Practice, 21, 103–119. 6988647 10.1111/j.1748-5827.1980.tb01221.x

[jsap70129-bib-0019] Jencikova‐Celerin, L. , Phillips, J.H. , Werk, L. , Wiltrout, S.A. & Nathanson, I. (2008) Flexible interlocked nailing of pediatric femoral fractures. Experience with a new flexible interlocking intramedullary nail compared with other fixation procedures. Journal of Pediatric Orthopaedics, 28, 864–873. 19034180 10.1097/BPO.0b013e31818e64a1

[jsap70129-bib-0020] Keeler, K.A. , Dart, B. , Luhmann, S.J. , Schoenecker, P.L. , Ortman, M.R. , Dobbs, M.B. et al. (2009) Antegrade intramedullary nailing of pediatric femoral fractures using an interlocking pediatric nail and a lateral trochanteric entry point. Journal of Pediatric Orthopaedics, 29, 345–351. 19461375 10.1097/BPO.0b013e3181a53b59

[jsap70129-bib-0021] Kolata, R.J. & Johnston, D.E. (1975) Motor vehicle accidents in urban dogs: a study of 600 cases. Journal of the American Veterinary Medical Association, 167, 938–941. 1184425

[jsap70129-bib-0022] Kolata, R.J. , Kraut, N.H. & Johnston, D.E. (1974) Patterns of trauma in urban dogs and cats: a study of 1,000 cases. Journal of the American Veterinary Medical Association, 164, 499–502. 4813404

[jsap70129-bib-0023] Kumar, H. , Tyagi, S.P. , Kumar, A. , Kumar, A. & Singh, S. (2020) Incidence of fractures in animals. Indian Journal of Veterinary Surgery, 41, 62–63.

[jsap70129-bib-0024] Lansdowne, J.L. , Sinnott, M.T. , Déjardin, L.M. , Ting, D. & Haut, R.C. (2007) In vitro mechanical comparison of screwed, bolted, and novel interlocking nail systems to buttress plate fixation in torsion and mediolateral bending. Veterinary Surgery, 36, 368–377. 17547600 10.1111/j.1532-950X.2007.00277.x

[jsap70129-bib-0025] Libardoni, R.N. , da Costa, D. , Menezes, F.B. , Cavalli, L.G. , Pedrotti, L.F. , Kohlrausch, P.R. et al. (2018) Classification, fixation techniques, complications and outcomes of femur fractures in dogs and cats: 61 cases (2015‐2016). Ciência Rural, 48, e20170028.

[jsap70129-bib-0026] Minar, M. , Hwang, Y. , Park, M. , Kim, S. , Oh, C. , Choi, S. et al. (2013) Retrospective study on fractures in dogs. Journal of Biomedical Research, 14, 140–144.

[jsap70129-bib-0027] Perry, K.L. & Wesslen, R. (2025) Outcomes of 243 dogs with traumatic fractures treated with the I‐loc interlocking nail. Veterinary Surgery, 54, 1549–1560. 40726100 10.1111/vsu.14320 PMC12618164

[jsap70129-bib-0028] Peterson, J.L. , Torres, B.T. , Hutcheson, K.D. & Fox, D.B. (2020) Radiographic determination of normal canine femoral alignment in the sagittal plane: a cadaveric pilot study. Veterinary Surgery, 49, 1230–1238. 32484579 10.1111/vsu.13465

[jsap70129-bib-0029] Phillips, I.R. (1979) A survey of bone fractures in the dog and cat. The Journal of Small Animal Practice, 20, 661–674. 547112 10.1111/j.1748-5827.1979.tb06679.x

[jsap70129-bib-0030] Raouf, M.A.E. , Ezzeldein, S.A. & Eisa, E.F.M. (2019) Bone fractures in dogs: a retrospective study of 129 dogs. Iraqi Journal of Veterinary Sciences, 33, 401–405.

[jsap70129-bib-0031] Roels, J. , Hebrard, L. , Saban, C. , Maggiar, A. , Ragetly, G. , Leperlier, D. et al. (2024) Retrospective study of the early clinical experience with a precountered angle‐stable interlocking nail for fracture repair in dogs and cats. American Journal of Veterinary Research, 85, 1–10. 38262138 10.2460/ajvr.23.09.0207

[jsap70129-bib-0032] Rumph, P.F. & Hathcock, J.T. (1990) A symmetric axis‐based method for measuring the projected femoral angle of inclination in dogs. Veterinary Surgery, 19, 328–333. 2219669 10.1111/j.1532-950x.1990.tb01200.x

[jsap70129-bib-0033] Sarrau, S. , Meige, F. & Autefage, A. (2007) Treatment of femoral and tibial fractures in puppies by elastic plate osteosynthesis. A review of 17 cases. Veterinary and Comparative Orthopaedics and Traumatology, 20, 51–58. 17364097

[jsap70129-bib-0034] Smith, B.A. , Kerwin, S.C. , Hosgood, G. , Voyiadjis, G. , Echle, R. & Strain, G.M. (1996) Mechanical comparison of two methods for interfragmentary fixation in a short oblique fracture model. Veterinary and Comparative Orthopaedics and Traumatology, 9, 145–151.

[jsap70129-bib-0035] Sutton, L.K. , Byrd, J.H. & Brooks, J.W. (2018) Age determination in dogs and cats. In: Brooks, J. (Ed.) Veterinary forensic pathology, Vol. 2. Cham: Springer.

[jsap70129-bib-0036] Talaat, A. , Shaaban, G. , Farghali, H.A. & Sharshar, A.M. (2022) Retrospective study on canine femoral fractures: incidence and surgical management. Journal of Current Veterinary Research, 4, 91–103.

[jsap70129-bib-0037] Tomlinson, J. , Fox, D. , Cook, J.L. & Keller, G.G. (2007) Measurement of femoral angles in four dog breeds. Veterinary Surgery, 36, 593–598. 17686134 10.1111/j.1532-950X.2007.00309.x

[jsap70129-bib-0038] Torzilli, P.A. , Takebe, K. , Burstein, A.H. & Heiple, K.G. (1981) Structure properties of immature canine bone. Journal of Biomechanical Engineering, 103, 232–238. 7311488 10.1115/1.3138286

[jsap70129-bib-0039] Torzilli, P.A. , Takebe, K. , Burstein, A.H. , Zika, J.M. & Heiple, K.G. (1982) The material properties of immature bone. Journal of Biomechanical Engineering, 104, 12–20. 7078113 10.1115/1.3138297

[jsap70129-bib-0040] Vaughan, L.C. (1975) Complications associated with the internal fixation of fractures in dogs. The Journal of Small Animal Practice, 16, 415–426. 1177419 10.1111/j.1748-5827.1975.tb05765.x

[jsap70129-bib-0041] Vizcaíno Revés, N. , Stahl, C. , Stoffel, M. , Bali, M. & Forterre, F. (2013) CT scan based determination of optimal bone corridor for atlantoaxial ventral screw fixation in miniature breed dogs. Veterinary Surgery, 42, 819–824. 24033689 10.1111/j.1532-950X.2013.12046.x

